# Multi-Pulsed High Hydrostatic Pressure Treatment of Foods

**DOI:** 10.3390/foods4020173

**Published:** 2015-05-25

**Authors:** Sencer Buzrul

**Affiliations:** Tütün ve Alkol Piyasası Düzenleme Kurumu (TAPDK), Ankara 06520, Turkey; E-Mail: sencer.buzrul@tapdk.gov.tr; Tel.: +90-312-218-0438; Fax: +90-312-218-0433

**Keywords:** high hydrostatic pressure, multi-pulsed high hydrostatic pressure, compression and decompression rates, microbial inactivation, pulse holding time

## Abstract

Multi-pulsed high hydrostatic pressure (mpHHP) treatment of foods has been investigated for more than two decades. It was reported that the mpHHP treatment, with few exceptions, is more effective than the classical or single-pulsed HHP (spHHP) treatment for inactivation of microorganisms in fruit juice, dairy products, liquid whole egg, meat products, and sea foods. Moreover, the mpHHP treatment could be also used to inactivate enzymes in foods and to increase the shelf-life of foods. The effects of the mpHHP treatment of foods are summarized and the differences between the mpHHP and spHHP are also emphasized.

## 1. Introduction

High hydrostatic pressure (HHP) treatment is an effective technique to destroy microorganisms and inactivate enzymes in order to enhance safety and shelf-life of foods. Therefore, HHP has become a reality in the food industry and has spread world wide [1]. After 2000, the number of installed HPP machines for the food industry increased exponentially [2].

**Figure 1:**
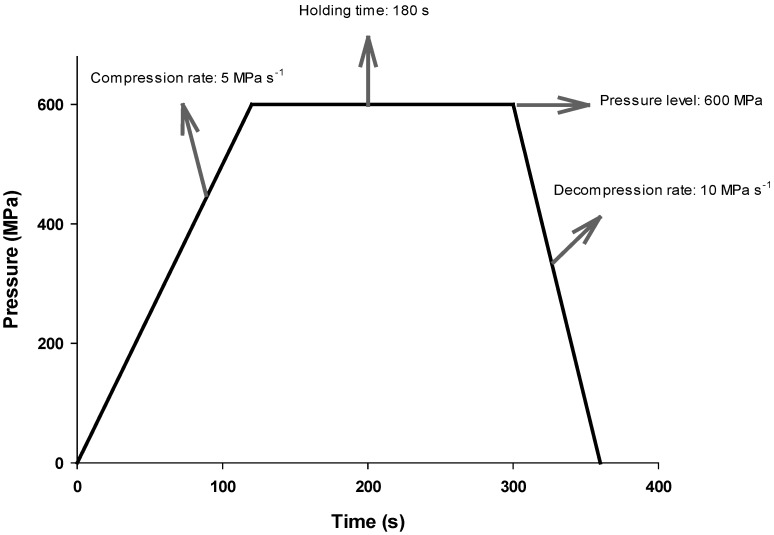
Classical or single-pulsed high hydrostatic pressure (HHP) treatment consisting of compression rate (5 MPa·s^−1^), holding time (180 s) at a constant pressure level (600 MPa), and decompression rate (10 MPa·s^−1^). Note that total duration of the treatment is 360 s (6 min).

**Figure 2:**
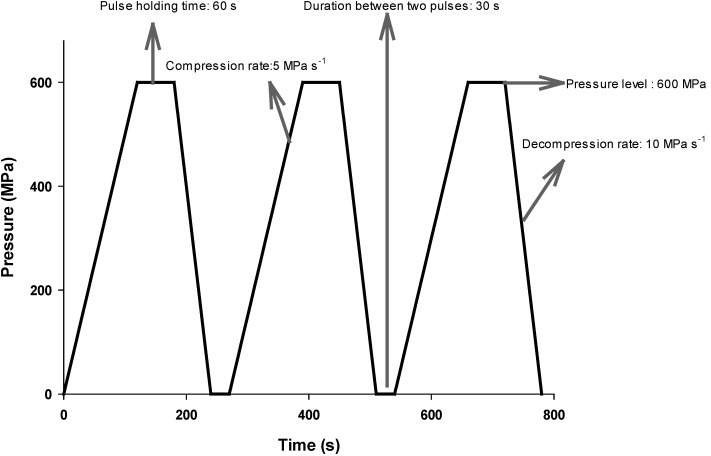
Multi-pulsed HHP treatment: 3 pulses × 60 s (30 s between each pulse) at 600 MPa. Compression and decompression rates are 5 and 10 MPa·s^−1^, respectively. Note that total duration of the treatment is 780 s (13 min).

Classical HHP or single-pulsed HHP (spHHP) treatment can be applied as: compression to target pressure, holding for a certain period of time at the target pressure, and decompression to atmospheric pressure (Figure 1). On the other hand, it may be also possible to apply successive application of HHP in which more than one compression, holding, and decompression periods exist (Figure 2). This type of treatment is called multi-pulsed HHP (mpHHP).

The mpHHP treatment, for the same holding time, is more effective than the spHHP treatment for enzyme [3,4,5], yeast cells [6] bacterial cells [7,8], and bacterial spores [9,10,11] inactivation. It was also reported that there was less recovery from injury for *Escherichia coli* for the mpHHP treatment compared to the spHHP treatment [12]. However, some researchers reported that the use of the mpHHP treatment did not considerably enhance pressure inactivation of virus [13], and bacteria [14,15] as compared to the sHHP treatment.

The mpHHP treatment inactivation of microorganisms in laboratory media, foods, blood plasma, vaccines, and drugs is well documented by a recent review [16]. This review, however, provides information not only about microorganisms but also enzymes, food quality and shelf-life.

## 2. Process Parameters of the mpHHP Treatment

It is known that pressure, temperature, and (holding) time are the most important process parameters of the spHHP treatment. However, more parameters should be taken into account before applying the mpHHP. Pressure and temperature are also the most important parameters for the mpHHP treatment. Besides, pulse duration, *i.e.*, pulse holding time, number of pulses, off-pressure time (duration between the pulses), compression and decompression rates or times, and pulse shape (ramp, square, sinusoidal) may also affect the outcomes of the mpHHP treatment (Figure 2). The effect of these process parameters on microbial inactivation was given by Buzrul [16] and will not be deeply investigated here.

## 3. Application of the mpHHP on Foods

### 3.1. Fruit Juices

Studies on fruit juices by the application of mpHHP began about two decades ago. The mpHHP treatment was reported to be more effective than the spHHP treatment for the same holding time for the inactivations of *Saccharomyces cerevisiae* in pineapple juice [17], *Byssochlamys nivea* ascospores in apple and cranberry juices [18]. On the other hand, Alemán *et al.* [17] observed no inactivation of *S. cerevisiae* in pineapple juice after 40–4000 fast sinusodial pulses (10 cycles/s) at 4–400 s total holding time in the range of 235–270 MPa (total processing time was 0.39–39 min) indicating that pulse shape is (step pressure pulse was effective, but sinusodial pulses had no effect on inactivation of yeasts in fruit juice) also an important parameter for the mpHHP treatment.

Donsì *et al.* [19] found that efficiency of the mpHHP treatment depends on the combination of pulse holding time and number of pulses for the inactivation of *S. cerevisiae* in pineapple and orange juices. They also observed higher reduction for slow compression rate (2.5 MPa·s^−1^) than that of faster compression rates (10.5 and 25 MPa·s^−1^) if several pulses (3 to 10 pulses) were applied. Buzrul *et al.* [20] found that increasing the pulse number did not effect the inactivation of *Escherichia coli* and *Listeria innocua* to great extends in kiwifruit juice (high inactivations were already obtained by application of the spHHP treatment in kiwifruit juice); however, in pineapple juice especially after 5 pulses inactivation increased significantly for both bacteria.

The mpHHP treatment up to 3 pulses with no holding time, *i.e.*, compression followed by decompression was also applied to inactivate pectin methyl esterase (PME) in single strength and concentrated orange juices [21]. The mpHHP has a significant contribution to inactivation of PME in both juices.

A comprehensive study by Donsì *et al.* [22] indicated that the effectiveness of the mpHHP treatment on apple and orange juices depends on the combination of pressure, temperature, and pulse number. Optimum conditions applied to apple (300 MPa, 50 °C, 6 pulses × 1 min) and orange (250 MPa, 45 °C, 6 pulses × 1 min) juices resulted in a minimum shelf-life of 21 days at 4 °C.

### 3.2. Dairy Products

Milk, cheese and yogurt are the dairy products treated with the mpHHP. The mpHHP was considerably more effective than the spHHP for the same total treatment time for inactivation of *E. coli* in skim milk [23], *E. coli* and *L. innocua* in whole milk [24,25].

The mpHHP treatment up to 4 pulses with no holding time was applied to inactivate *E. coli* O157:H7 and *L. monocytogenes* in raw milk cheese [26]. Significant microbial and enzyme inactivation could be possible by the application of the mpHHP (at room temperature, 3 pulses × 5 min) at higher pressures (600 and 800 MPa) in three different types of cheese which were at different ripening stages [27]. Storage of cheeses at 5 °C for 12 weeks revealed that microorganisms inactivated by the mpHHP were also absent during storage. López-Pedemonte *et al.* [28] obtained low inactivation (about 1.6 log_10_) for spores of *Bacillus cereus* in cheese by the mpHHP inactivation with 2 pulses (first pulse with low pressure (60 MPa) to germinate the spores and the second one is with high pressure (400 MPa) to inactivate the vegetative cells).

Applications of the spHHP (400 MPa for 15, 30, and 45 min) and mpHHP (400 MPa for 3 pulses × 5 min, 3 pulses × 10 min, and 3 pulses × 15 min) treatments in yogurt revealed that *Lactobacillus delbruecki* sp. *bulgaricus* was completely inactivated under all conditions whereas *Streptoccocus salivarius* sp. *thermophilus* was little reduced, maximum by one log_10_ [29].

### 3.3. Liquid Whole Egg

A few studies on microbial inactivation in liquid whole egg (LWE) by the mpHHP treatment revealed that the mpHHP treatment showed greater effectiveness than the spHHP treatment for inactivations of *Samonella* Enteritidis [30,31,32] and *E. coli* [33].

The effect of temperature during the mpHHP treatment is well documented in these studies. For example, Ponce *et al.* [33] applied the spHHP (350 MPa, 10 or 15 min) and the mpHHP (350 MPa, 2 or 3 pulses × 5 min) treatments at different temperatures (2, 20, or 50 °C) for the inactivation of *E. coli* in LWE. The highest reduction was achieved at 50 °C for both treatments; however, at lower temperatures, especially at 20 °C, the mpHHP treatment was clearly more effective than the spHHP treatments. Ponce *et al.* [30] observed the strongest effectiveness at 50 °C, followed by 20, 2, and −15 °C for the inactivation of *S.* Enteritidis in LWE after the application of mpHHP (2 or 3 pulses × 5 min at 350 and 450 MPa).

### 3.4. Meat Products

The mpHHP applied to mechanically recovered poultry meat showed that the mpHHP treatment was slightly better than the spHHP treatment for psychrotrophs, but the mpHHP treatment did not offer better results than the spHHP treatment for mesophiles [15,34]. On the other hand, the use of the mpHHP treatment instead of the spHHP treatment showed to be more advantageous for the inactivation of *E. coli* O157:H7 in ground beef [35] and *S.* Enteritidis in chicken breast fillets especially at higher pressures [36].

Morales *et al.* [35] and Del Olmo *et al.* [37] studied the effect of the spHHP and mpHHP treatments on color and texture of beef patties and chicken breast fillets, respectively. Changes in the color and texture of ground beef caused by spHHP and mpHHP treatments of the same lethality for *E. coli* O157:H7 (20 min for spHHP and 4 pulses × 1 min for mpHHP) were similar [35]. Color parameters (*L* *, *a* * and *b* *) were significantly higher for both treatments than for vacuum-packaged control fillets. Similarly, the texture of chicken breast fillets was also significantly affected by both treatments [37].

### 3.5. Sea Foods

The effect of the spHHP (400 MPa, 7 °C, 10 min) and the mpHHP (400 MPa, 7 °C, 2 pulses × 5 min) on microbial flora, total volatile bases, pH, and texture of purified and unpurified oysters was studied by López-Caballero *et al.* [14]. The mpHHP produced no apparent advantages over the spHHP based on any of the indices used.

The mpHHP treatment reduced the microbial load in octopus arm muscle more effectively than the spHHP treatment; however, the mpHHP treatment was not so effective in reducing autolytic activity [38,39]. Inactivations of *S.* Enteritidis and *Staphyloccoccus aureus* in sturgeon and trout caviar also studied [40]. Results indicated that the mpHHP treatment (350 MPa for *S.* Enteritidis and 450 MPa for *Staphyloccoccus aureus* at room temperature for 3 pulses × 5 min) were as effective as the spHHP treatment (400 MPa for *S.* Enteritidis and 500 MPa for *Staphyloccoccus aureus* at room temperature for 15 min).

### 3.6. Other Food Products

Similar results were also obtained for other food products: the mpHHP treatment was more effective than the spHHP treatment for the inactivation of *S. cerevisiae* in fresh cut pineapple [41], *S.* Enteritidis in raw almonds [42], *E. coli* in egg white [43]. Meyer [44] reported sterility in macaroni and cheese with spore load of *Clostridium sporogenes* and *B. cereus* by the mpHHP treatment (690 MPa, 90 °C, 2 pulses × 1 min; 1 min pause between the pulses).

A summary of the mpHHP inactivation of microorganisms in foods and a summary of studies on the effect of mpHHP treatment on quality, shelf-life, microbial and enzyme inactivation of foods are provided in Table 1 and Table 2, respectively.

**Table 1:** Summary of multi-pulsed high hydrostatic pressure (mpHHP) inactivation of microorganisms in foods.

Microorganism	Product	CR or CT ^a^	DR or DT ^b^	Process Conditions ^c^	Log Reduction	Reference
*Saccharomyces cerevisiae*	Pineapple juice	0.5 s	0.2 s	270 MPa, 23 °C, 10 pulses × 10 s	3.3	[17]
				270 MPa, 23 °C, 100 pulses × 1 s	3.5	
		0.34 s	0.18 s	270 MPa, 23 °C, 167 pulses × 0.6 s	3.9	
				(0.2 s between the pulses)		
*Byssochlamys nivea*	Cranberry juice	2.4 MPa·s^−1^	<10 s	689 MPa, 60 °C, 3 pulses × 1 s	>4.0 *	[18]
*ascospores*	Apple juice			689 MPa, 60 °C, 3 pulses × 1 s	>4.0 *	
*S. cerevisiae*	Pineapple juice	10.5 MPa·s^−1^	ND ^d^	250 MPa, 25 °C, 10 pulses × 1 min	4.0	[19]
	Orange juice			250 MPa, 25 °C, 6 pulses × 1 min	>4.5	
				250 MPa, 25 °C, 10 pulses × 1 min	>5.0	
				200 MPa, 45 °C, 6 pulses × 1 min	>5.0	
				200 MPa, 45 °C, 10 pulses × 1 min	≈5.5	
		2.5 MPa·s^−1^	ND	200 MPa, 25 °C, 10 pulses × 1 min	≈2.7	
		25 MPa·s^−1^	ND	200 MPa, 25 °C, 10 pulses × 1 min	≈2.2	
*Escherichia coli*	Pineapple juice	5 MPa·s^−1^	5 MPa·s^−1^	300 MPa, 20 °C, 10 pulses × 30 s	2.8	[20]
				350 MPa, 20 °C, 5 pulses × 60 s	2.6	
*Listeria innocua*				300 MPa, 20 °C, 10 pulses × 30 s	3.4	[20]
				350 MPa, 20 °C, 5 pulses × 60 s	3.6	
*E. coli*	Kiwifruit juice			300 MPa, 20 °C, 10 pulses × 30 s	4.7	[20]
				350 MPa, 20 °C, 5 pulses × 60 s	5.5	
*L. innocua*				300 MPa, 20 °C, 10 pulses × 30 s	4.8	[20]
				350 MPa, 20 °C, 5 pulses × 60 s	5.6	
*E. coli*	Skim milk	ND	ND	550 MPa, 20 °C, 3 pulses × 10 min	6.0	[23]
	Whole milk	5 MPa·s^−1^	5 MPa·s^−1^	400 MPa, 20–25 °C, 10 pulses × 1 min	4.0	[25]
				400 MPa, 20–25 °C, 10 pulses × 2 min	4.6	
*L. innocua*				400 MPa, 20–25 °C, 5 pulses × 4 min	3.9	[25]
				400 MPa, 20–25 °C, 10 pulses × 2 min	4.3	
*E. coli* K-12	Raw milk cheese	2.25 MPa·s^−1^	< 3s	400 MPa, 25 °C, 4 pulses × 0 min	≈3.4	[26]
*E. coli* O157:H7				400 MPa, 25 °C, 4 pulses × 0 min	≈1.4	[26]
*L. monocytogenes*				400 MPa, 25 °C, 4 pulses × 0 min	≈3.8	[26]
*Bacillus cereus spores*	Cheese	ND	ND	60 MPa, 30 °C, 210 min + 400 MPa	1.6	[28]
				30 °C, 15 min		
*S*. Enteritidis	Liquid whole egg	180 s	90 s	350 MPa, 50 °C, 2 pulses × 5 min	7.8 *	[30]
		240 s	120 s	450 MPa, 20 °C, 2 pulses × 5 min	7.3 *	
		ND	ND	138 MPa, 20 °C, 2 pulses × 4 min	1.3	[31]
		45 s	6 s	350 MPa, 50 °C, 4 pulses × 2 min	>8.0 *	[32]
*E. coli* O157:H7	Ground beef	2.2 min	0.3 min	400 MPa, 12 °C, 3 pulses × 5 min	≈3.0	[35]
*S*. Enteritidis	Chicken breast	96 s	16.2 s	300 MPa, 12 °C, 2 pulses × 5 min	2.5	[36]
	fillets	132 s	19.2 s	400 MPa, 12 °C, 3 pulses × 3 min	4.6	
*Enterobacteriaceae*	Octopus muscle	4 min	≈2 s	400 MPa, 7 °C, 3 pulses × 5 min	≈3.0	[39]
				400 MPa, 40 °C, 3 pulses × 5 min	≈3.0	
*S*. Enteritidis	Sturgeon caviar	ND	ND	450 MPa, 20 °C, 3 pulses × 5 min	>4.1	[40]
	Trout caviar			450 MPa, 20 °C, 3 pulses × 5 min	>2.7	
*S. aureus*	Sturgeon caviar	ND	ND	450 MPa, 20 °C, 3 pulses × 5 min	>3.5	[40]
	Trout caviar			450 MPa, 20 °C, 3 pulses × 5 min	>3.7	
*S*. Enteriditis	Raw almonds	≈3.6 min	1 min	414 MPa, 50 °C, 6 pulses × 20 s	1.3	[42]
				(30 s between the pulses)		
*E. coli*	Egg white	ND	ND	300 MPa, 20 °C, 3 pulses × 2 min	>7.0 *	[43]
*B. cereus*	Macaroni and	ND	ND	690 MPa, 90 °C, 2 pulses × 1 min	>6.0 *	[44]
*spores*	cheese			(1 min between the pulses)		
*Clostridium sporogenes* spores				690 MPa, 90 °C, 2 pulses × 1 min	> 6.0 *	[44]
				(1 min between the pulses)		

^a^ CR: Compression rate; CT: Compression time; ^b^ DR: Decompression rate; DT: Decompression time; ^c^ The temperature given is either the initial or the process temperature of the treatment; ^d^ ND: Not determined; * Total inactivation.

**Table 2:** Summary of studies on the effect of mpHHP treatment on quality, shelf-life, microbial and enzyme inactivation of foods.

Product	CR or CT ^a^	DR or DT ^b^	Process conditions ^c^	Achievement	Reference
Oyster	2.5 MPa·s^−1^	15 s	400 MPa, 7 °C, 2 pulses × 5 min	No apparent advantages over	[14]
				spHHP treatment	
Orange juice	2.8 min	≈10 s	400 MPa, 20 °C, 3 pulses × 0 s	92.4% inactivation of PME	[21]
	10.5 MPa·s^−1^	4 s	250 MPa, 45 °C, 6 pulses × 60 s	21 days of shelf-life at 4 °C	[22]
Apple juice	10.5 MPa·s^−1^	5 s	300 MPa, 50 °C, 6 pulses × 60 s	21 days of shelf-life at 4 °C	
Cheese	ND ^d^	ND	800 MPa, ND, 3 pulses × 5 min	4–6 log_10_ inactivation of microorganisms	[27]
				Inactivation of proteases	
				No growth of inactivated	
				microorganisms at 5 °C for 12 weeks	
Yogurt	ND ^d^	ND	400 MPa, ND, 3 pulses × 5 min	Complete inactivation of	[29]
				*Lactobacillus bulgaricus*	
				No acidity change at 1 and 20 °C	
				for 3 weeks	
Ground beef	2.2 min	0.3 min	400 MPa, 12 °C, 2 pulses × 60 s	Significant color and texture changes	[35]
Chicken breast fillets	2.2 min	17 s	400 MPa, 5 °C, 2 pulses × 60 s	Significant color and texture changes	[37]

^a^ CR: Compression rate; CT: Compression time; ^b^ DR: Decompression rate; DT: Decompression time; ^c^ The temperature given is either the initial or the process temperature of the treatment; ^d^ ND: Not determined.

## 4. Commercial Application of the mpHHP

Although there is now enough evidence that the mpHHP treatment is an effective way of inactivating microorganisms and enzymes, there is no commercial application of the mpHHP treatment up to date. One and most important reason for this is that the mpHHP treatment is a longer application and thus more expensive than the spHHP treatment [8,13,16,25,41]—see Figure 1 and Figure 2. Besides, most probably a more complicated HHP equipment which can withstand fast compression and decompression rates (to reduce the total duration of the treatment) is needed for commercial applications.

However, as the technology improves it may be possible to have faster compression and decompression rates hence it may be possible to reach compatible total treatment times for the commercial applications of the mpHHP treatment. To the best of the author’s knowledge there is no study on the cost and optimization of process parameters of the mpHHP treatment. Optimization between pulse number, pulse holding time, pressure level, compression and decompression rates as well as initial and target temperature in a lab scale equipment will accelerate the application of commercial mpHHP treatment. Moreover, the differences in effectiveness of mpHHP and spHHP treatments must be weighed against the design capabilities of added wear on the HHP equipment, and possible additional time required for pulse treatment [45].

## 5. Conclusions

The mpHHP treatment could be used to inactivate microorganisms and enzymes in foods. It could also be used to contribute the quality and shelf-life of foods. However, it should be noted that optimization between the pressure, temperature, pulse number, pulse holding time, and compression and decompression rates can increase the effectiveness of the mpHHP treatment. However, more studies are needed especially on the cost of the mpHHP treatment.

## References

[1] Bermúdez-Aguirre D., Barbosa-Cánovas G.V. (2011). An update on high hydrostatic pressure, from laboratory to industrial applications. Food Eng. Rev..

[2] Tonello C., Zhang H.Q., Barbosa-Cánovas G.V., Balasubramaniam V.M., Dunne C.P., Farkas D.F., Yuan J.T.C. (2011). Case studies on high-pressure processing of foods. Nonthermal Processing Technologies for Food.

[3] Curl A.L., Jansen E.F. (1949). Effect of high pressures on trypsin and chymotrypsin. J. Biol. Chem..

[4] Curl A.L., Jansen E.F. (1950). The effect of high pressures on pepsin and chymotrypsinogen. J. Biol. Chem..

[5] Ludikhuyze L.R., van den Broeck I., Weemaes C.A., Hendrickx M.E. (1997). Kinetic parameters for pressuretemperature inactivation of *Bacillus subtilis* α-amylase under dynamic conditions. Biotechnol. Prog..

[6] Palou E., López-Malo A., Barbosa-Cánovas G.V., Welti-Chanes J., Swanson B.G. (1998). Oscillatory high hydrostatic pressure inactivation of *Zygosaccharomyces bailii*. J. Food Prot..

[7] Masschalck B., García-Graells C., van Haver E., Michiels C.W. (2000). Inactivation of high pressure resistant *Escherichia coli* by lysozyme and nişin under high pressure. Innov. Food Sci. Emerg. Technol..

[8] Rivalain N., Roquain J., Boiron J.M., Maurel J.P., Largeteau A., Ivanovic Z., Demazeau G. (2012). High hydrostatic pressure treatment for the inactivation of *Staphylococcus aureus* in human blood plasma. New Biotechnol..

[9] Hayakawa I., Kanno T., Yoshiyama K., Fujio Y. (1994). Oscillatory compared with continuous high pressure sterilization on *Bacillus stearothermophilus* spores. J. Food Sci..

[10] Furukawa S., Nakahara A., Hayakawa I. (2000). Effect of reciprocal pressurization on germination and killing of bacterial spores. Int. J. Food Sci. Technol..

[11] Ahn J., Balasubramaniam V.M. (2007). Effects of inoculum level and pressure pulse on the inactivation of *Clostridium sporogenes* spores by pressure-assisted thermal processing. J. Microbiol. Biotechnol..

[12] Pilavtepe-Çelik M., Buzrul S., Alpas H., Largeteau A., Demazeau G. (2011). Multi-pulsed high hydrostatic pressure treatment for inactivation and injury of *Escherichia Coli*. J. Verbrauch. Lebensm..

[13] Kingsley D.H., Guan D., Hoover D.G., Chen H. (2006). Inactivation of Hepatitis A virus by high-pressure processing: The role of temperature and pressure oscillation. J. Food Prot..

[14] López-Caballero M.E., Pérez-Mateos M., Montero P., Borderías A.J. (2000). Oyster preservation by high-pressure treatment. J. Food Prot..

[15] Yuste J., Pla R., Capellas M., Sendra E., Beltran E., Mor-Mur M. (2001). Oscillatory high pressure processing applied to mechanically recovered poultry meat for bacterial inactivation. J. Food Sci..

[16] Buzrul S. (2014). Multi-pulsed high hydrostatic pressure treatment of microorganisms: A review. Innov. Food Sci. Emerg. Technol..

[17] Alemán G.D., Ting E.Y., Mordre S.C., Hawes A.C.O., Walker M., Farkas D.F., Torres J.A. (1996). Pulsed ultra high pressure treatments for pasteurization of pineapple juice. J. Food Sci..

[18] Palou E., López-Malo A., Barbosa-Cánovas G.V., Welti-Chanes J., Davidson P.M., Swanson B.G. (1998). Effect of oscillatory high hydrostatic pressure treatments on *Byssochlamys nivea* ascospores suspended in fruit juice concentrates. Lett. Appl. Microbiol..

[19] Donsì G., Ferrari G., Maresca P. (2007). Pulsed high pressure treatment of *Saccharomyces cerevisiae*: The effect of process parameters. J. Food Eng..

[20] Buzrul S., Largeteau A., Alpas H., Demazeau G. (2008). Inactivation of *Escherichia coli* and *Listeria innocua* in kiwifruit and pineapple juices by high hydrostatic pressure. Int. J. Food Microbiol..

[21] Basak S., Ramaswamy H.S. (2001). Pulsed high pressure inactivation of pectin methyl esterase in single strength and concentrated orange juices. Can. Biosyst. Eng..

[22] Donsì G., Ferrari G., Maresca P. (2010). Pasteurization of fruit juices by means of a pulsed high pressure process. J. Food Sci..

[23] García-Graells C., Masschalck N., Moonjai N., Michiels C., Ludwig H. (1999). High pressure inactivation and survival of pressure-resistant *Escherichia coli* mutants in milk. Advances in High Pressure Bioscience and Biotechnology.

[24] Buzrul S., Largeteau A., Alpas H., Demazeau G. (2008). Pulsed pressure treatment for inactivation of *Escherichia coli* and *Listeria innocua* in whole milk. J. Phys. Conf. Ser..

[25] Buzrul S., Alpas H., Largeteau A., Demazeau G. (2009). Efficiency of pulse pressure treatment for inactivation of *Escherichia coli* and *Listeria innocua* in whole milk. Eur. Food Res. Technol..

[26] Shao Y., Ramaswamy H.S., Zhu S. (2007). High-pressure destruction kinetics of spoilage and pathogenic bacteria in raw milk cheese. J. Food Proc. Eng..

[27] Reps A., Kołakowski P., Dajnowiec F., Isaacs N.S. (1998). The effect of high pressure on microorganisms and enzymes of ripening cheeses. High Pressure Food Science, Bioscience & Chemistry.

[28] López-Pedemonte T.J., Roig-Sagués A.X., Trujillo A.J., Capellas M., Guamis B. (2003). Inactivation of spores of *Bacillus cereus* in cheese by high hydrostatic pressure with the addition of nisin or lysozyme. J. Dairy Sci..

[29] Reps A., Warminska-Radyko I., Dajnowiec F., Ludwig H. (1999). Effect of high pressure on yogurt. Advances in High Pressure Bioscience and Biotechnology.

[30] Ponce E., Pla R., Sendra E., Guamis B., Mor-Mur M. (1999). Destruction of *Salmonella enteritidis* inoculated in liquid whole egg by high hydrostatic pressure: Comprative study in selective and non-selective media. Food Microbiol..

[31] Huang E., Mittal G.S., Griffiths M.W. (2006). Inactivation of Salmonella enteritidis in liquid whole egg using combination of pulsed electrical field, high pressure and ultrasound. Biosyst. Eng..

[32] Bari M.L., Ukuku D.O., Mori M., Kawamoto S., Yamamoto K. (2008). Effect of hydrostatic pressure pulsing on the inactivation of *Salmonella* Enteritidis in liquid whole egg. Foodborne Path. Dis..

[33] Ponce E., Pla R., Capellas M., Guamis B., Mor-Mur M. (1998). Inactivation of *Escherichia coli* inoculated in liquid whole egg by high hydrostatic pressure. Food Microbiol..

[34] Yuste J., Mor-Mur M., Capellas M., Guamis B., Pla R. (1998). Microbiological quality of mechanically recovered poultry meat treated with high hydrostatic pressure and nisin. Food Microbiol..

[35] Morales P., Calzada J., Ávila M., Nuñez M. (2008). Inactivation of *Escherichia coli* O157:H7 in ground beef by single-cycle and multiple-cycle high-pressure treatments. J. Food Prot..

[36] Morales P., Calzada J., Rodríguez B., de Paz M., Nuñez M. (2009). Inactivation of *Salmonella* Enteritidis in chicken breast fillets by single-cycle and multiple-cycle high pressure treatments. Foodborne Path. Dis..

[37] Del Olmo A., Morales P., Ávila M., Calzada J., Nuñez M. (2010). Effect of single-cycle and multiple-cycle high-pressure treatments on the colour and texture of chicken breast fillets. Innov. Food Sci. Emerg. Technol..

[38] Hurtado J.L., Montero P., Borderías A.J., Ludwig H. (1999). Influence of high isostatic pressure on muscle of octopus (*Octopus vulgaris*). Advances in High Pressure Bioscience and Biotechnology.

[39] Hurtado J.L., Montero P., Borderías J., Solas M. (2001). High-pressure/temperature treatment effect on the characteristics of octopus (*Octopus vulgaris*) arm muscle. Eur. Food Res. Technol..

[40] Fioretto F., Cruz C., Largeteau A., Sarli T.A., Demazeau G., El Moueffak A. (2005). Inactivation of *Staphylococcus aureus* and *Salmonella enteritidis* in tryptic soy broth and caviar samples by high pressure processing. Braz. J. Med. Biol. Res..

[41] Alemán G.D., Ting E.Y., Farkas D.F., Mordre S.C., Hawes A.C.O., Torres J.A. (1998). Comparison of static and step-pulsed ultra-high pressure on the microbial stability of fresh cut pineapple. J. Sci. Food Agric..

[42] Goodridge L.D., Willford J., Kalchayanand N. (2006). Destruction of *Salmonella* Enteriditis inoculated onto raw almonds by high hydrostatic pressure. Food Res. Int..

[43] Homma K., Haga N., Hayashi R. (1990). Effect of high pressure-treatment on sterilization and physical properties of egg white. High Pressure Science for Food.

[44] Meyer R. (2000). Ultra High Pressure, High Temperature Food Preservation Process.

[45] Farkas D.R., Hoover D.G. (2000). High pressure processing. J. Food Sci..

